# Supplementary figures and images for: Hypersensitive intercellular responses of endometrial stromal cells drive invasion in endometriosis
Source: eLife. 2024 Dec 11;13:e94778. doi: 10.7554/eLife.94778 (PMC11729374; doi:10.7554/eLife.94778)

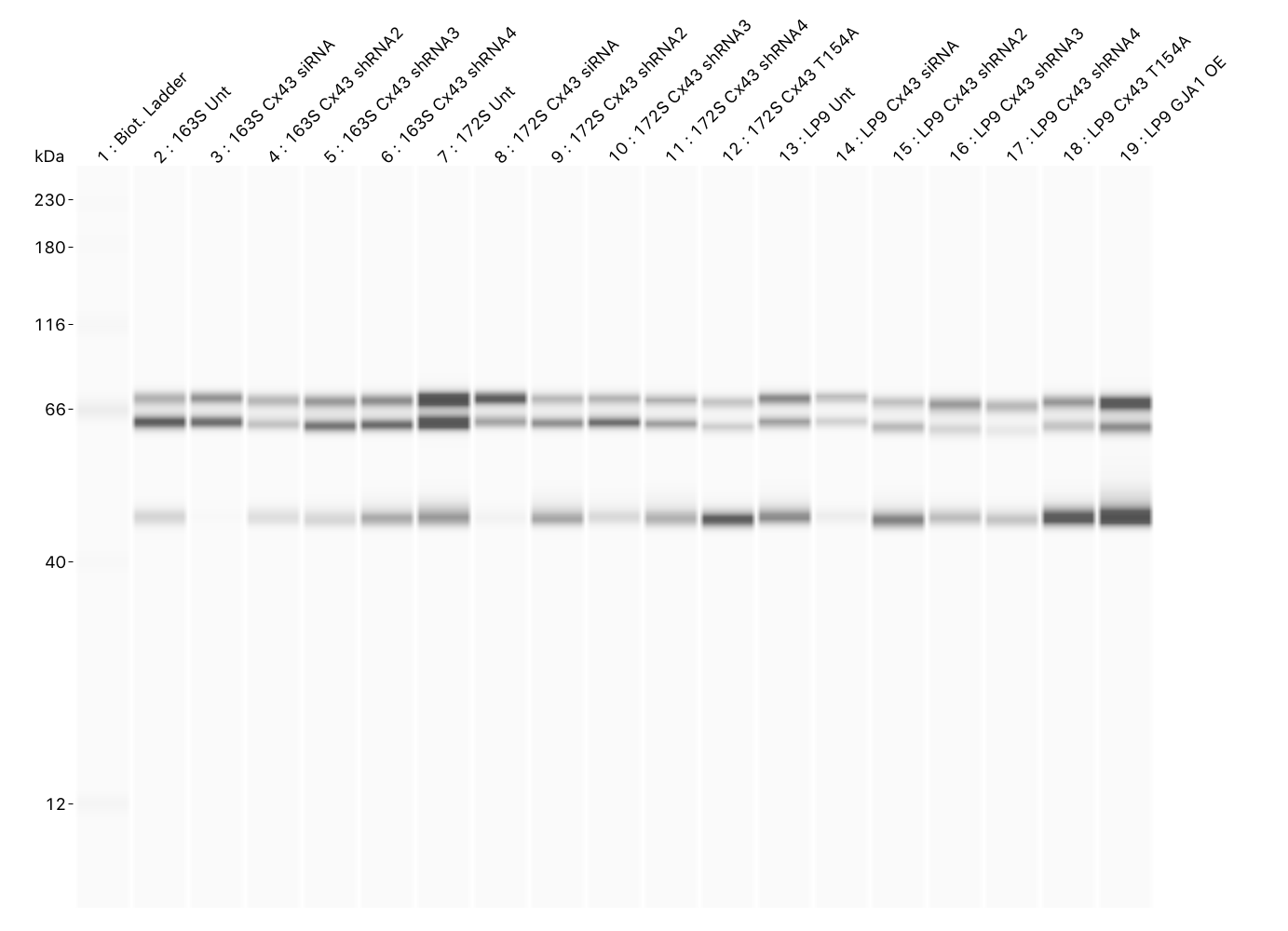

Supplement: Figure 4—source data 1. [file elife-94778-fig4-data1.zip › Figure 4 - source data 1 - gel image for 4B.tiff]

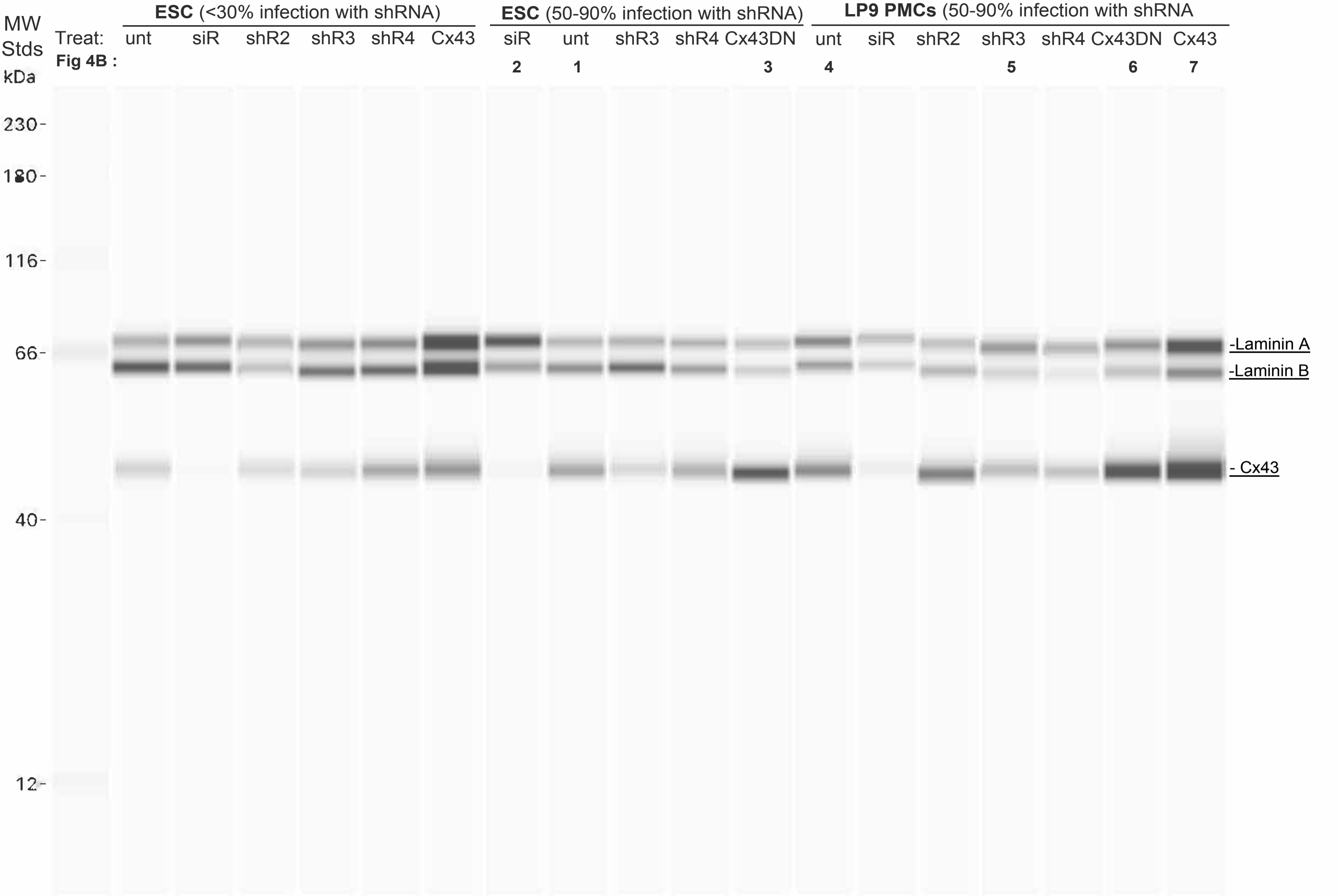

Supplement: Figure 4—source data 2. [file elife-94778-fig4-data2.pdf]
